# Prognostic value of nine inflammatory biomarkers for critically ill patients with rheumatic heart disease: a retrospective study

**DOI:** 10.3389/fimmu.2025.1610967

**Published:** 2025-05-29

**Authors:** Ying Zhang, Xiaofei Ni

**Affiliations:** Department of Emergency, The Affiliated Zhangjiagang Hospital of Soochow University, Suzhou, China

**Keywords:** critical care, inflammatory biomarker, mortality, platelet-to-lymphocyte ratio, rheumatic heart disease

## Abstract

**Background:**

Among various inflammatory biomarkers, the prognostic value in critically ill patients with rheumatic heart disease (RHD) remains unclear. This study aimed to compare the prognostic value of different inflammatory biomarkers in patients with RHD.

**Methods:**

This study identified critically ill patients admitted to the intensive care unit from the Medical Information Mart for Intensive Care IV database (MIMIC-IV). Nine systemic inflammatory biomarkers, derived from various combinations of neutrophils, lymphocytes, monocytes, and platelets, were evaluated for their association with 30-day all-cause mortality. Receiver operating characteristic curve analysis was performed to identify the most predictive biomarker. Furthermore, Cox proportional hazards regression and restricted cubic spline analysis were employed to evaluate the association between the optimal biomarker and survival outcomes.

**Results:**

A total of 1002 patients with RHD were included. Eight inflammatory biomarkers were predictive for 30-day all-cause mortality and the platelet-to-lymphocyte ratio (PLR) demonstrated the highest area under the curve value of 0.794 among these biomarkers. Then patients were divided into tertiles based on PLR. Multivariate Cox proportional hazards analysis demonstrated that an elevated PLR was significantly associated with increased 30-day all-cause mortality. After adjustment for potential confounders, elevated PLR remained an independent predictor of mortality (adjusted hazard ratio: 2.53; 95% confidence interval: 1.87–3.42; p < 0.001). Furthermore, restricted cubic spline analysis revealed a progressively increasing risk of all-cause mortality with higher PLR levels.

**Conclusion:**

These findings indicate that the PLR may be a useful indicator for evaluating the severity and guiding the treatment of RDH patients.

## Introduction

Rheumatic heart disease (RHD) is an autoimmune disorder triggered by infection with group A Streptococcus (GAS), typically following acute rheumatic fever (ARF), and is characterized by progressive damage to the heart valves (1). It is the most common form of heart disease among children and young adults, with a global burden exceeding 40 million cases and causing approximately 306,000 deaths annually (2). The morbidity and mortality associated with RHD complications pose a significant challenge, particularly in resource-limited healthcare setting (3, 4). Therefore, early recognition and diagnosis are essential to guide timely and appropriate interventions, ultimately improving patient outcomes and survival.

Recently, growing efforts have been made to identify reliable biomarkers for RHD. Among autoantibodies, IgG2 specific to N-acetyl-β-D-glucosamine (GlcNAc) has been proposed as an early marker for acute rheumatic fever (ARF) and a contributor to RHD pathogenesis, with elevated serum levels and valvular deposition observed in RHD patients (5). Circular RNAs (circRNAs) have also shown promise in RHD. Zhu et al. reported that hsa_circ_0000437 is upregulated in patients with rheumatic valvular heart disease (RVHD), promoting cell proliferation and migration while inhibiting apoptosis. Similarly, hsa_circ_0003748 was found to regulate human valvular interstitial cells through miR-577 sponging (6). Hematologic parameters such as red cell distribution width (RDW), platelet distribution width (PDW), mean platelet volume (MPV), and C-reactive protein (CRP) were significantly elevated in pediatric RHD patients compared to healthy controls, with RDW correlating positively with chronic inflammation (7). Inflammatory cytokines also play a key role in RHD prognosis. Elevated levels of IL-1β, IL-6, IL-8, CXCL-1, and TNF-α have been associated with disease severity (8, 9). One study found higher cytokine levels in severe versus stable RHD cases, and identified IL-6/TNF-α and IL-6/IL-17A ratios as effective severity indicators. IL-10 and IL-4 were also independent predictors of adverse outcomes (10).

These findings highlight the value of biomarkers in RHD diagnosis and prognosis, with inflammatory markers showing particular promise. The relationship between inflammatory biomarkers based on blood cell count and mortality in RHD patients remains unexplored. This study aims to compare the prognostic significance of multiple inflammatory biomarkers in patients with RHD.

## Materials and methods

### Patients selection

This retrospective study used clinical data from the Medical Information Mart for Intensive Care IV database (MIMIC-IV) (version 2.0) database, a publicly accessible and well-validated critical care resource developed by the Massachusetts Institute of Technology Laboratory for Computational Physiology. MIMIC-IV contains comprehensive, high-quality medical records of patients in intensive care units at Beth Israel Deaconess Medical Center (11). Ying Zhang (ID: 14346643) registered, completed training, and received permission to access the MIMIC-IV database. Informed consent was unnecessary for data collection since all patient data in the database is anonymized to maintain privacy and confidentiality.

Patients with RDH were identified in the MIMIC-IV database using International Classification of Diseases version 9 (ICD-9) and ICD-10 diagnostic codes. Patients with RHD were identified using ICD-9 codes 9-391, 9-393, 9-394, 9-395, 9-3979, 9-3980, 9–39890 and ICD-10 codes 10-I01, 10-I05, 10-I06, 10-I07, 10-I08. Exclusion criteria included: (1) patients younger than 18 years at initial admission; (2) those diagnosed with end-stage renal disease, liver cirrhosis, or cancer; (3) ICU stays shorter than 24 hours; and (4) missing survival data.

### Data collection

Data extraction utilized PostgreSQL 13.7.2 and Navicat Premium 16 with structured query language. The extracted variables were organized into four primary categories: (1) patient demographics, encompassing age and gender; (2) clinical severity scores, such as the Oxford Acute Severity of Illness Score (OASIS), Glasgow Coma Scale (GCS), and Charlson Comorbidity Index (CCI); (3) comorbidities, including respiratory failure, diabetes, hypertension, renal disease, sepsis, and paraplegia; (4) laboratory parameters at Intensive Care Unit (ICU) admission, comprising neutrophils, lymphocytes, monocytes, and platelets counts (**Supplementary Figure S1**). Follow-up commenced on the date of ICU admission and continued until the date of death.

Nine inflammatory biomarkers were calculated using routine peripheral blood counts. The neutrophil–monocyte index (NM) was defined as neutrophil count × monocyte count; the neutrophil–platelet index (NP) as neutrophil count × platelet count; and the monocyte–platelet index (MP) as monocyte count × platelet count. The neutrophil-to-lymphocyte ratio (NLR), monocyte-to-lymphocyte ratio (MLR), and platelet-to-lymphocyte ratio (PLR) were calculated by dividing the respective cell counts by the lymphocyte count. The systemic immune-inflammation index (SII) was defined as platelet count × neutrophil count/lymphocyte count, and the systemic inflammation response index (SIRI) as neutrophil count × monocyte count/lymphocyte count. The aggregate index of systemic inflammation response (AIRI) was calculated as platelet count × neutrophil count × monocyte count/lymphocyte count (**Supplementary Table S1**).

The primary outcomes of this study were 30-day ICU all-cause mortality. To ensure data completeness, cases missing three or more of the four key blood cell counts (neutrophils, lymphocytes, monocytes, and platelets) at ICU admission were excluded. Variables with over 20% missing data were excluded from the analysis to minimize potential bias. Variables with less than 20% missing data were imputed using a random forest algorithm through the ‘mice’ package in R software (12) (**Supplementary Table S2**).

### Statistical analysis

The Shapiro-Wilk test was used to assess the normality of continuous variables, revealing that most did not follow a normal distribution. Continuous variables were expressed as medians with interquartile ranges and compared between groups using the Wilcoxon rank-sum test. Categorical variables were expressed as frequencies and percentages, and group comparisons were performed using the Chi-square test. Receiver operating characteristic (ROC) curves were generated for each inflammatory biomarker to evaluate their predictive performance for 30-day all-cause mortality, with the optimal cut-off value determined using the Youden index. Comparisons between two ROC curves were performed using both the DeLong test (13). Kaplan–Meier survival curves were used to compare 30-day ICU survival between groups, with the log-rank test evaluating differences. Cox proportional hazards regression models assessed the relationship among different groups, presenting findings as hazard ratios (HRs) with 95% confidence intervals (CIs). The proportional hazards assumption was tested using Schoenfeld residuals function. Clinically relevant and prognosis-associated variables were included in the multivariate Cox regression models. Model 1 was unadjusted; Model 2 was adjusted for age and gender; Model 3 was adjusted for age, gender, OASIS, GCS, and CCI; and Model 4 was further adjusted for age, gender, OASIS, GCS, CCI, respiratory failure, diabetes, hypertension, renal disease, sepsis, and paraplegia. Additionally, a restricted cubic spline regression model with three knots was used to explore the potential nonlinear association between the baseline optimal biomarker and 30-day ICU all-cause mortality. Subgroup analyses were conducted to examine the association between optimal biomarker and mortality within predefined categories such as age, gender, respiratory failure, diabetes, hypertension, renal disease, sepsis, and paraplegia. A two-sided p-value < 0.05 was considered statistically significant. Analyses were performed using R software (v4.2.1).

## Results

### Patient characteristics

A total of 1002 critically ill patients diagnosed with RHD were included in the study. The median age was 73.39 years (interquartile range [IQR], 64.43–80.65), with 488 (48.7%) females and 514 (51.3%) males. The 30-day ICU mortality rates were 6.19%. Compared with survivors, non-survivors were significantly older (median: 77.20 vs. 73.09 years, p < 0.001) and had higher severity scores, including OASIS and CCI (p < 0.001 for both). A higher proportion of non-survivors had respiratory failure (37.1% vs. 21.1%, p = 0.007), diabetes (48.4% vs. 30.9%, p = 0.008), renal disease (43.6% vs. 27.6%, p = 0.012), and sepsis (40.3% vs. 12.0%, p < 0.001), while no significant differences were observed in hypertension or paraplegia. Regarding laboratory parameters, non-survivors had significantly lower lymphocyte counts and higher inflammatory indices such as NLR, PLR, SII, SIRI, and AIRI (all p < 0.05). Platelet counts, NP, and MP were also significantly elevated in the non-survivor group. The median length of stay (LOS) in ICU for patients was 2.46 days (IQR: 1.36–4.91 days). Length of ICU stay was longer in non-survivors compared to survivors (median: 4.73 vs. 2.42 days, p = 0.007). The clinicopathological characteristics of the patients included in this study are presented in **Table 1**.

**Table 1:** Baseline characteristics of the non-survivors and survivors groups.

Variables	Total (n=1002)	Non-survivor (n=62)	Survivor (n=940)	p-value
Personal characteristics				
Age (years)	73.39 (64.43,80.65)	77.20 (69.98,84.10)	73.09 (63.98,80.40)	<0.001
Gender (%)				0.941
Female	488 (48.70)	31 (50.00)	457 (48.62)	
Male	514 (51.30)	31 (50.00)	483 (51.38)	
Scores				
OASIS	32.00 (26.00,37.75)	38.00 (33.00,46.00)	32.00 (26.00,37.00)	<0.001
GCS	15.00 (14.00,15.00)	15.00 (13.00,15.00)	15.00 (14.00,15.00)	0.062
CCI	5.00 (3.00,7.00)	7.00 (5.25,8.75)	5.00 (3.00,7.00)	<0.001
Comorbidities				
Respiratory failure (%)				0.007
No	781 (77.94)	39 (62.90)	742 (78.94)	
Yes	221 (22.06)	23 (37.10)	198 (21.06)	
Diabetes (%)				0.008
No	682 (68.06)	32 (51.61)	650 (69.15)	
Yes	320 (31.94)	30 (48.39)	290 (30.85)	
Hypertension (%)				0.425
No	989 (98.70)	60 (96.77)	929 (98.83)	
Yes	13 (1.30)	2 (3.23)	11 (1.17)	
Renal disease (%)				0.012
No	716 (71.46)	35 (56.45)	681 (72.45)	
Yes	286 (28.54)	27 (43.55)	259 (27.55)	
Sepsis (%)				<0.001
No	864 (86.23)	37 (59.68)	827 (87.98)	
Yes	138 (13.77)	25 (40.32)	113 (12.02)	
Paraplegia (%)				0.932
No	976 (97.41)	61 (98.39)	915 (97.34)	
Yes	26 (2.59)	1 (1.61)	25 (2.66)	
Laboratory tests				
Lymphocytes (10^9^/L)	1.62 (0.98,2.39)	0.70 (0.40,1.18)	1.67 (1.04,2.44)	<0.001
Monocytes (10^9^/L)	0.60 (0.36,0.93)	0.59 (0.23,0.93)	0.60 (0.36,0.93)	0.576
Neutrophils (10^9^/L)	9.68 (7.05,13.72)	9.49 (6.05,13.48)	9.74 (7.08,13.73)	0.772
Platelets (10^9^/L)	156.00 (120.00,194.00)	193.50 (131.00,251.02)	155.00 (120.00,192.00)	0.005
NM	5.44 (2.75,10.89)	7.87 (2.92,13.92)	5.33 (2.74,10.52)	0.621
NP	1456.72 (948.52,2291.34)	2008.94 (1111.82,3903.29)	1435.10 (945.45,2230.96)	0.027
MP	88.25 (46.51,165.51)	123.83 (52.84,232.37)	86.07 (46.04,160.19)	0.034
NLR	5.91 (3.63,10.13)	12.26 (6.87,23.28)	5.64 (3.56,9.76)	<0.001
MLR	0.36 (0.18,0.71)	0.72 (0.26,1.49)	0.35 (0.18,0.66)	0.006
PLR	4.52 (4.06,5.12)	5.55 (4.99,6.07)	4.49 (4.04,5.04)	<0.001
SII	860.36 (525.36,1649.49)	2328.75 (1012.34,4509.52)	836.90 (515.60,1550.23)	0.008
SIRI	3.25 (1.51,7.65)	6.54 (1.55,15.01)	3.09 (1.51,7.30)	0.024
AIRI	505.50 (201.98,1327.01)	1198.44 (242.76,3232.62)	481.04 (201.78,1216.42)	0.022
Outcomes				
ICU LOS (days)	2.46 (1.36,4.91)	4.73 (1.47,10.42)	2.42 (1.36,4.46)	0.007

Medians and interquartile ranges (25th and 75th percentiles) were calculated for continuous variables and frequencies and percentages for categorical variables. The Wilcoxon rank sum test was used to compare group differences for continuous variables and Chiiablesss tests for categorical variables.

AIRI, aggregate index of systemic inflammation response; CCI, charlson comorbidity index; GCS, glasgow coma scale; ICU, intensive care unit; LOS, length of stay; MLR, monocyte-to-lymphocyte ratio; MP, monocyte × platelet; NLR, neutrophil-to-lymphocyte ratio; NM, neutrophil × monocyte; NP, neutrophil × platelet; OASIS, oxford acute severity of illness score; PLR, platelet-to-lymphocyte ratio; SII, systemic immune-inflammation index; SIRI, systemic inflammation response index.

### ROC curves for 30-day all-cause mortality

**Figure 1:**
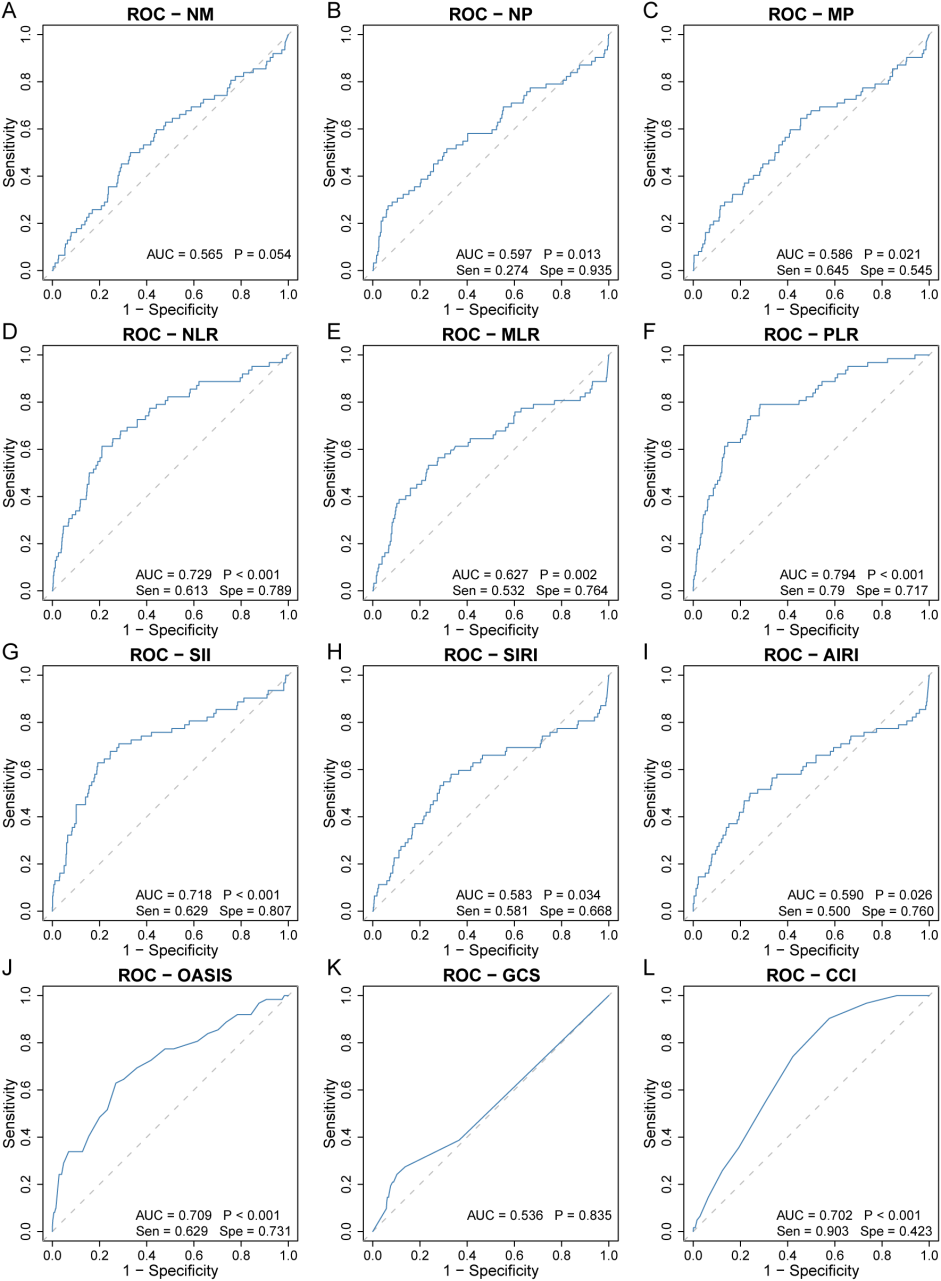
Receiver operating characteristic curve analysis to evaluate the predictive value of nine inflammatory biomarkers for 30-day ICU all-cause mortality in rheumatic heart disease patients. **(A)** NM, **(B)** NP, **(C)** MP, **(D)** NLR, **(E)** MLR, **(F)** PLR, **(G)** SII, **(H)** SIRI, **(I)** AIRI, **(J)** OASIS, **(K)** GCS, **(L)** CCI. AIRI, aggregate index of systemic inflammation response; AUC, area under the curve; CCI, charlson comorbidity index; GCS, glasgow coma scale; MLR, monocyte-to-lymphocyte ratio; MP, monocyte × platelet; NLR, neutrophil-to-lymphocyte ratio; NM, neutrophil × monocyte; NP, neutrophil × platelet; OASIS, oxford acute severity of illness score; PLR, platelet-to-lymphocyte ratio; ROC, receiver operating characteristic; SII, systemic immune-inflammation index; Sen, sensitivity; SIRI, systemic inflammation response index; Spe, specificity.

**Figure 2:**
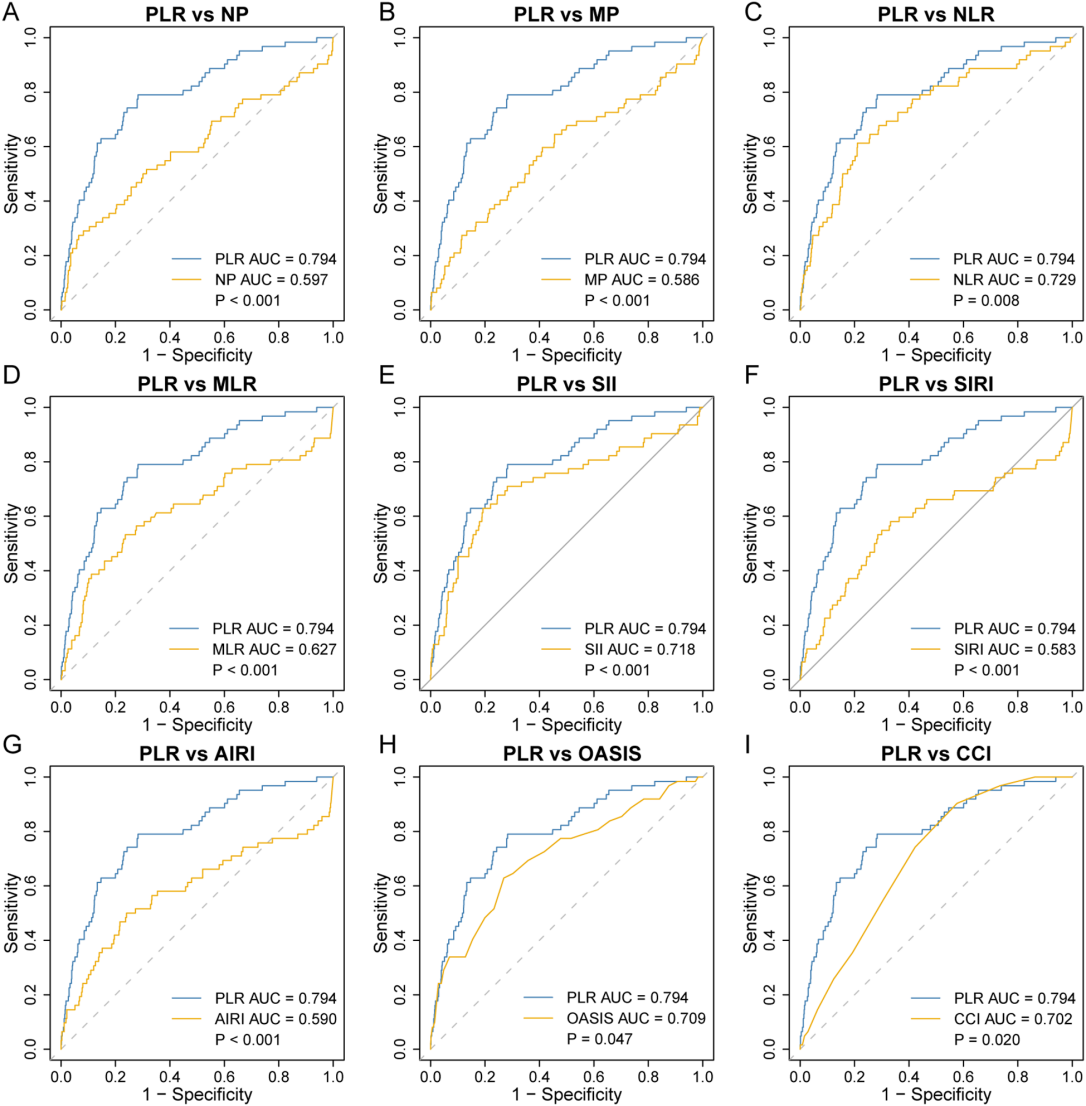
Comparison of receiver operating characteristic curve analysis for 30-day ICU all-cause mortality in rheumatic heart disease patients. PLR was compared to NP **(A)**, MP **(B)**, NLR **(C)**, MLR **(D)**, SII **(E)**, SIRI **(F)**, AIRI **(G)**, OASIS **(H)**, and CCI **(I)**. AIRI, aggregate index of systemic inflammation response; AUC, area under the curve; CCI, charlson comorbidity index; MLR, monocyte-to-lymphocyte ratio; MP, monocyte × platelet; NLR, neutrophil-to-lymphocyte ratio; NP, neutrophil × platelet; OASIS, oxford acute severity of illness score; PLR, platelet-to-lymphocyte ratio; SII, systemic immune-inflammation index; SIRI, systemic inflammation response index.

Among the nine inflammatory biomarkers, eight (NP, MP, NLR, MLR, PLR, SII, SIRI, and AIRI) showed significantly higher area under the curve (AUC) values (p < 0.05) for predicting 30-day ICU all-cause mortality (**Figures 1A–I**), with PLR exhibiting the highest AUC (0.794). We then compared the AUC of PLR with those of the other seven biomarkers using the DeLong test. PLR demonstrated significantly higher predictive accuracy than NP (p < 0.001), MP (p < 0.001), NLR (p = 0.008), MLR (p < 0.001), SII (p < 0.001), SIRI (p < 0.001), and AIRI (p < 0.001) (**Figures 2A–G**). In addition, we evaluated the prognostic performance of OASIS, GCS, and CCI in predicting 30-day mortality. Both OASIS and CCI showed significantly elevated AUC values (p < 0.05) for 30-day ICU mortality among RHD patients (**Figures 1J–L**). When compared with PLR, the AUC for PLR was significantly higher than that of OASIS (p = 0.047) and CCI (p = 0.020) (**Figures 2H, I**). Subsequent analyses focused on PLR. To normalize its distribution and reduce skewness, PLR values were log-transformed prior to regression modeling.

### Correlation between clinicopathological factors and PLR

The baseline characteristics of critically ill patients with RHD, stratified by PLR tertiles, are presented in **Table 2**. Patients were divided into three groups based on PLR levels at ICU admission: tertile 1 (T1, n = 333; PLR:1.62-70.10), tertile 2 (T2, n = 333; PLR: 70.10-134.29), and tertile 3 (T3, n = 336; PLR: 134.29-2,418.94). The median PLR values for each tertile were 48.91(36.99, 57.82) in T1, 91.80 (80.75, 109.02) in T2, and 232.74 (167.94, 333.86) in T3. Significant differences were observed among the three groups. Patients in the highest PLR group (T3) were older and had higher CCI scores compared to those in T1 and T2 (p < 0.001). The proportions of respiratory failure, renal disease, and sepsis increased progressively across the tertiles (all p < 0.001). Laboratory results showed that lymphocyte counts decreased, while neutrophil, monocyte, and platelet counts increased with higher PLR levels (all p < 0.001). Composite inflammatory indicators including NLR, MLR, SII, SIRI, and AIRI were significantly elevated in the T3 group (p < 0.001). Notably, the 30-day ICU mortality was 1.20 percent in T1, 2.70 percent in T2, and 14.58 percent in T3 (p < 0.001).

**Table 2:** Characteristics and outcomes of participants categorized according to PLR tertiles.

Variables	PLR T1 (n=333)	PLR T2 (n=333)	PLR T3 (n=336)	p-value
PLR	48.91 (36.99, 57.82)	91.80 (80.75, 109.02)	232.74 (167.94, 333.86)	<0.001
Personal characteristics				
Age (years)	71.68 (62.69,78.74)	71.24 (63.97,78.65)	77.48 (67.21,85.11)	<0.001
Gender (%)				0.251
Female	152 (45.65)	161 (48.35)	175 (52.08)	
Male	181 (54.35)	172 (51.65)	161 (47.92)	
Scores				
OASIS	32.00 (26.00,37.00)	32.00 (26.00,37.00)	33.00 (27.00,39.00)	0.133
GCS	15.00 (14.00,15.00)	15.00 (14.00,15.00)	15.00 (14.00,15.00)	0.584
CCI	4.00 (3.00,6.00)	5.00 (3.00,7.00)	6.00 (5.00,8.00)	<0.001
Comorbidities				
Respiratory failure (%)				<0.001
No	285 (85.59)	254 (76.28)	242 (72.02)	
Yes	48 (14.41)	79 (23.72)	94 (27.98)	
Diabetes (%)				0.232
No	238 (71.47)	224 (67.27)	220 (65.48)	
Yes	95 (28.53)	109 (32.73)	116 (34.52)	
Hypertension (%)				0.591
No	330 (99.10)	329 (98.80)	330 (98.21)	
Yes	3 (0.90)	4 (1.20)	6 (1.79)	
Renal disease (%)				<0.001
No	270 (81.08)	239 (71.77)	207 (61.61)	
Yes	63 (18.92)	94 (28.23)	129 (38.39)	
Sepsis (%)				<0.001
No	320 (96.10)	297 (89.19)	247 (73.51)	
Yes	13 (3.90)	36 (10.81)	89 (26.49)	
Paraplegia (%)				0.962
No	324 (97.30)	325 (97.60)	327 (97.32)	
Yes	9 (2.70)	8 (2.40)	9 (2.68)	
Laboratory tests				
Lymphocytes (10^9^/L)	2.63 (2.17,3.51)	1.66 (1.34,2.05)	0.81 (0.57,1.08)	<0.001
Monocytes (10^9^/L)	0.56 (0.35,0.88)	0.56 (0.33,0.86)	0.70 (0.40,1.07)	0.008
Neutrophils (10^9^/L)	10.49 (7.98,14.28)	8.89 (6.49,12.93)	9.49 (6.69,13.80)	<0.001
Platelets (10^9^/L)	125.00 (102.00,153.00)	157.00 (127.00,186.33)	193.72 (155.75,242.25)	<0.001
NM	5.41 (2.95,10.40)	4.76 (2.37,9.04)	6.50 (3.18,12.71)	<0.001
NP	1302.14 (892.70,1936.40)	1341.90 (904.20,2107.28)	1867.86 (1141.35,2995.47)	<0.001
MP	66.40 (38.16,114.70)	80.85 (44.67,143.28)	144.23 (68.81,223.14)	<0.001
NLR	3.75 (2.78,5.10)	5.71 (3.80,7.77)	11.49 (7.96,20.07)	<0.001
MLR	0.21 (0.13,0.31)	0.35 (0.19,0.51)	0.84 (0.46,1.41)	<0.001
SII	482.66 (336.37,675.60)	821.86 (611.51,1198.62)	2241.60 (1345.33,3987.76)	<0.001
SIRI	2.10 (1.14,3.77)	2.81 (1.45,5.91)	7.61 (3.44,16.17)	<0.001
AIRI	256.00 (128.19,477.01)	444.15 (237.56,894.27)	1479.03 (642.50,3370.15)	<0.001
Outcomes				
ICU LOS (days)	2.51 (1.40,4.87)	2.31 (1.33,4.33)	2.59 (1.33,5.16)	0.201
30‐day ICU mortality (%)				<0.001
Alive	329 (98.80)	324 (97.30)	287 (85.42)	
Death	4 (1.20)	9 (2.70)	49 (14.58)	

Medians and interquartile ranges (25th and 75th percentiles) were calculated for continuous variables and frequencies and percentages for categorical variables. The Wilcoxon rank sum test was used to compare group differences for continuous variables and Chiiablesss tests for categorical variables.

AIRI, aggregate index of systemic inflammation response; CCI, charlson comorbidity index; GCS, glasgow coma scale; ICU, intensive care unit; LOS, length of stay; MLR, monocyte-to-lymphocyte ratio; MP, monocyte × platelet; NLR, neutrophil-to-lymphocyte ratio; NM, neutrophil × monocyte; NP, neutrophil × platelet; OASIS, oxford acute severity of illness score; PLR, platelet-to-lymphocyte ratio; T, tertile; SII, systemic immune-inflammation index; SIRI, systemic inflammation response index.

### Primary outcomes

**Figure 3:**
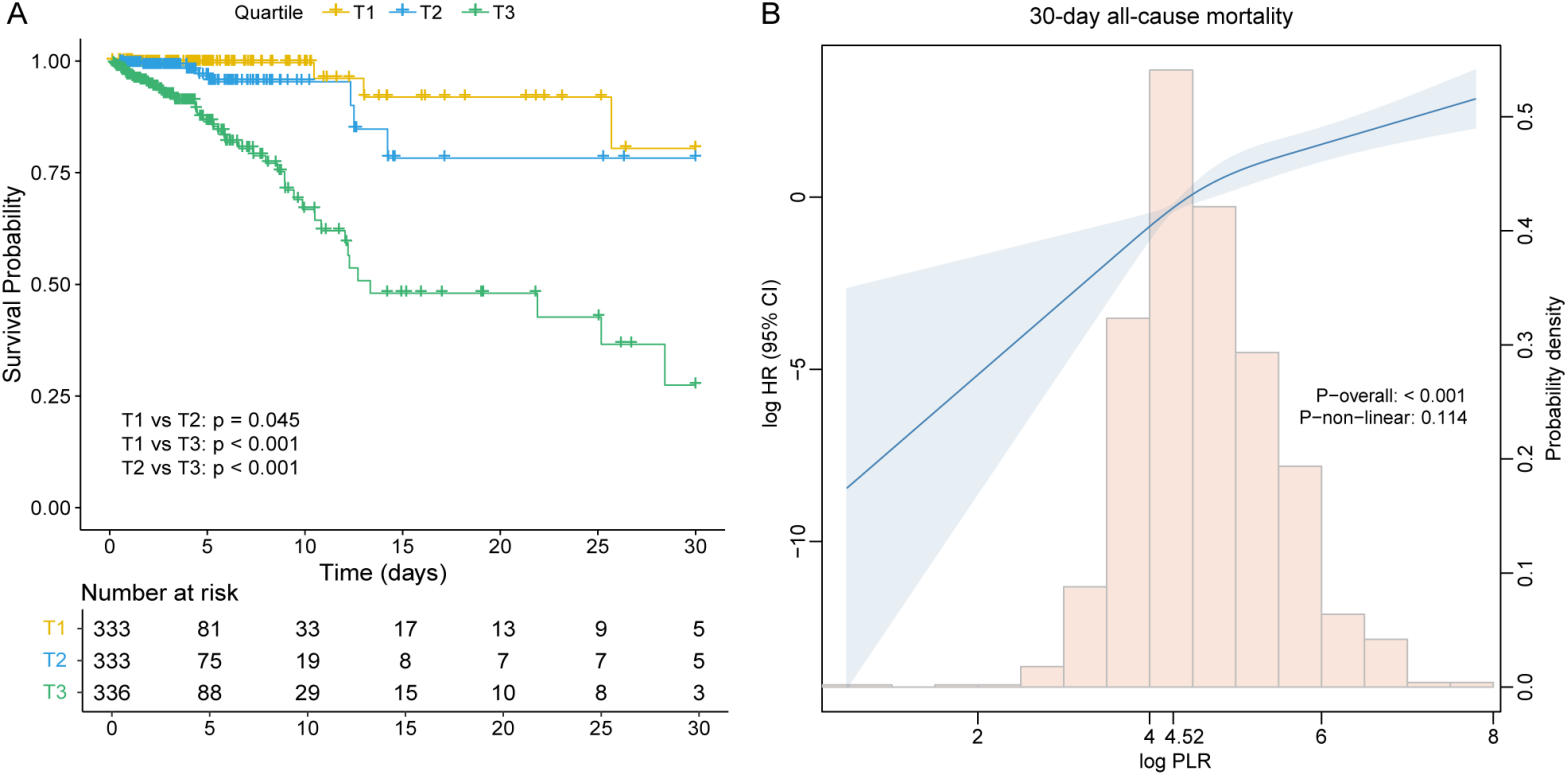
**(A)** Kaplan–Meier survival curves for 30-day all-cause mortality across PLR tertile groups. **(B)** Restricted cubic spline curve with 30-day all-cause mortality for the PLR hazard ratio. CI, confidence interval; HR, hazard ratio; PLR, platelet-to-lymphocyte ratio; T, tertile.

Kaplan–Meier survival analysis revealed significant differences in 30-day ICU survival among the three PLR tertiles (**Figure 3A**). Patients in the highest PLR group (T3) exhibited the poorest survival probability, while those in the lowest PLR group (T1) had the most favorable outcomes. The survival differences between all pairwise comparisons were statistically significant, with p = 0.045 for T1 versus T2, p < 0.001 for T1 versus T3, and p < 0.001 for T2 versus T3.

The proportional hazards assumption was verified using Schoenfeld residuals, and no significant violations were observed for any covariate or the global test (all p > 0.05) (**Supplementary Table S3**). Cox regression models demonstrated a strong positive association between PLR and mortality risk among critically ill patients with RHD. When considered as a continuous variable, PLR was consistently correlated with an increased risk of 30-day all-cause mortality across all models. In the fully adjusted model (Model 4), each unit increase in PLR was associated with a 2.53-fold increase in the risk of death (HR = 2.53, 95% CI: 1.87-3.42, p < 0.001). When analyzed as tertiles, patients in the highest PLR group (T3) had a substantially elevated risk compared to those in the lowest tertile (T1). The adjusted hazard ratios (HR) for T2 and T3 in Model 4 were 2.60 (95% confidence Interval[CI]: 1.01-8.62, p = 0.049) and 11.85 (95% CI: 3.98-35.24, p < 0.001), respectively, with a significant linear trend across tertiles (p for trend < 0.001). These associations remained robust after adjusting for demographic characteristics, severity scores, and comorbidities (**Table 3**), suggesting that PLR is an independent predictor of short-term mortality in RHD patients.

**Table 3:** Cox proportional hazard ratios for 30-day all-cause mortality in rheumatic heart disease patients.

Categories	Model 1		Model 2		Model 3		Model 4	
	HR (95%CI)	p-value	HR (95%CI)	p-value	HR (95%CI)	p-value	HR (95%CI)	p-value
30‐day all-cause mortality (Continuous variable)	2.59 (2.05,3.27)	<0.001	2.39 (1.87,3.07)	<0.001	2.60 (1.97,3.43)	<0.001	2.53 (1.87,3.42)	<0.001
PLR tertile								
T1	Ref		Ref		Ref		Ref	
T2	2.72 (1.02, 8.73)	0.047	2.89 (1.05, 9.41)	0.043	2.79 (1.05, 9.16)	0.044	2.60 (1.01, 8.62)	0.049
T3	12.69 (4.58,35.18)	<0.001	11.79 (4.24,32.78)	<0.001	12.86 (4.54,36.41)	<0.001	11.85 (3.98,35.24)	<0.001
HR for trend	5.18 (3.07,8.76)		4.73 (2.80,8.00)		5.15 (2.99,8.87)		4.97 (2.76, 8.93)	
p-value for trend		<0.001		<0.001		<0.001		<0.001

Model 1: unadjusted.

Model 2: adjusted for age, gender.

Model 3: adjusted for age, gender, OASIS, GCS, CCI.

Model 4: adjusted for age, gender, OASIS, GCS, CCI, respiratory failure, diabetes, hypertension, renal disease, sepsis, paraplegia.

AIRI, aggregate index of systemic inflammation response; CCI, charlson comorbidity index; CI, confidence Interval; GCS, glasgow coma scale; HR, hazard ratio; ICU, intensive care unit; LOS, length of stay; MLR, monocyte-to-lymphocyte ratio; MP, monocyte × platelet; NLR, neutrophil-to-lymphocyte ratio; NM, neutrophil × monocyte; NP, neutrophil × platelet; OASIS, oxford acute severity of illness score; PLR, platelet-to-lymphocyte ratio; T, tertile; Ref, reference; SII, systemic immune-inflammation index; SIRI, systemic inflammation response index.

### The detection of linear relationships

Restricted cubic spline (RCS) analysis revealed a positive, approximately linear relationship between PLR and 30-day all-cause mortality risk in patients with rheumatic heart disease, without significant evidence of non-linearity (p for non-linear = 0.114) (**Figure 3B**). To further explore the potential threshold effect, a two-piecewise Cox proportional hazards regression model was applied. The inflection point was identified at a PLR value of 4.52. Below this threshold, the risk of 30-day mortality increased steeply (HR = 8.64, 95% CI: 1.05-99.45, p = 0.047), while above the threshold, the association remained statistically significant but less pronounced (HR = 2.35, 95% CI: 1.69-3.26, p < 0.001) (**Table 4**). However, the log-likelihood ratio test comparing the one-line and two-line models did not indicate a significantly better fit for the two-piecewise model (p = 0.366), supporting the plausibility of a linear dose-response relationship between PLR and 30-day mortality.

**Table 4:** Threshold effect analysis of PLR on 30-day all-cause mortality in rheumatic heart disease patients.

30-day mortality	HR (95% CI)	p-value
Fitting by the standard linear regression	2.59 (2.05,3.27)	<0.001
Fitting model by two-piecewise linear regression		
Inflection point	4.52	
Log PLR < 4.52	8.64 (1.05,99.45)	0.047
Log PLR ≥ 4.52	2.35 (1.69,3.26)	<0.001
p for Log-likelihood ratio		0.366

CI, confidence Interval; HR, hazard ratio; PLR, platelet-to-lymphocyte ratio.

### Subgroup analysis

**Figure 4:**
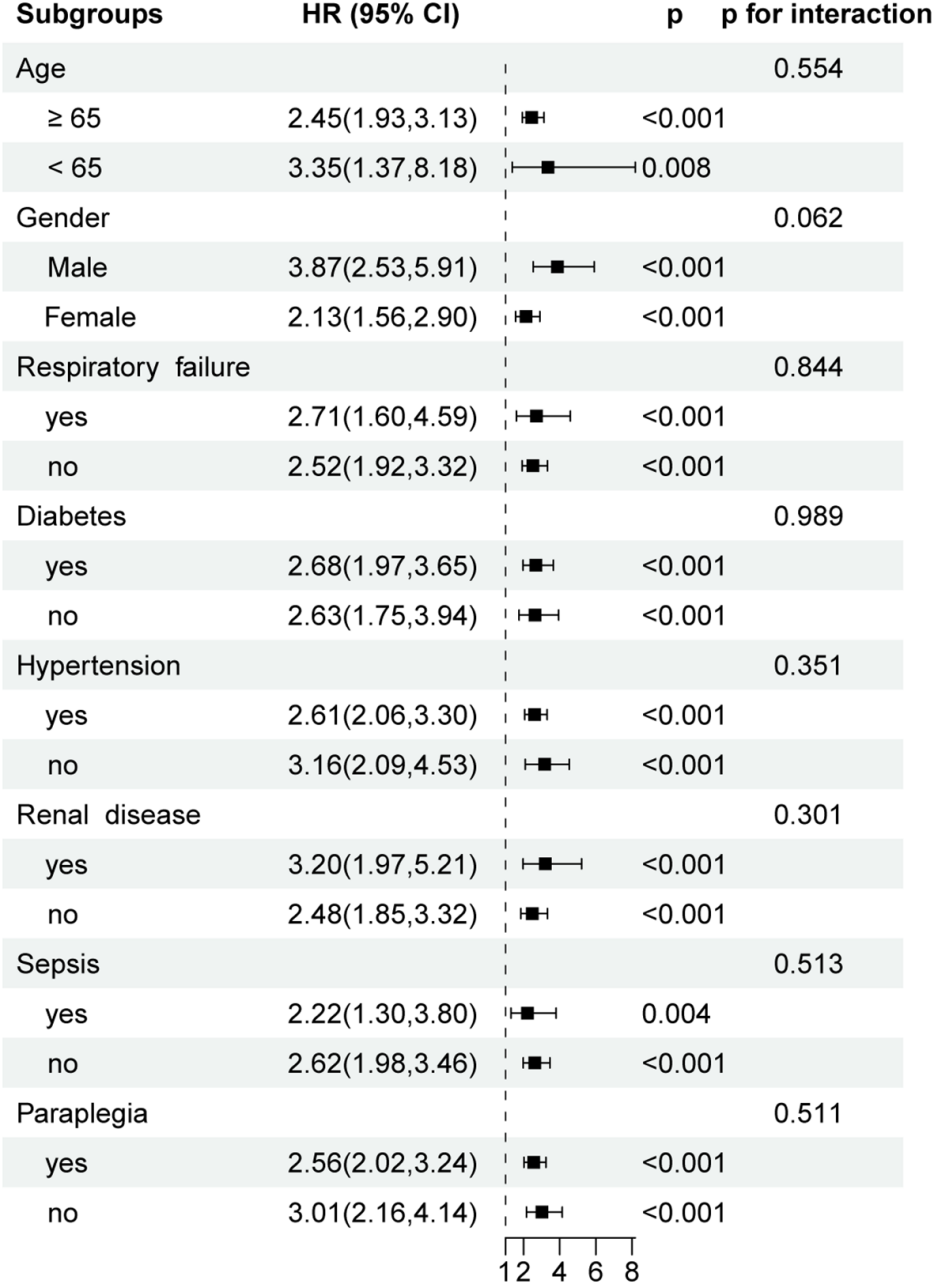
Forest plots of hazard ratios for 30-day ICU mortality in different subgroups. HR, hazard ratio; CI, confidence interval.

To assess the consistency of the association between PLR and 30-day all-cause mortality across clinically relevant subpopulations, stratified subgroup analyses were conducted based on age, gender, respiratory failure, diabetes, hypertension, renal disease, sepsis, and paraplegia status (**Figure 4**). Elevated PLR remained significantly associated with increased 30-day mortality in all subgroups analyzed (all p < 0.01). Specifically, HR for patients aged < 65 years was 3.35 (95% CI: 1.37-8.18, p = 0.008), while for those ≥ 65 years, the HR was 2.45 (95% CI: 1.93-3.13, p < 0.001), with no significant interaction observed (p for interaction = 0.554). Similarly, the association was present in both males (HR = 3.87, 95% CI: 2.53-5.91) and females (HR = 2.13, 95% CI: 1.56-2.90). The strength of association remained robust in subgroups with or without respiratory failure, diabetes, renal disease, or sepsis, and no significant effect modification was identified across any of the tested covariates (all p for interaction > 0.05).

## Discussion

In this retrospective study, we comprehensively evaluated the prognostic value of nine systemic inflammatory biomarkers in critically ill patients with RHD using the MIMIC-IV database. Among them, the PLR demonstrated the highest predictive performance for 30-day all-cause mortality, as indicated by the largest area under the ROC curve. Multivariate Cox regression analysis confirmed that elevated PLR was independently associated with increased mortality risk, and this association remained robust after adjusting for severity scores and comorbidities. Restricted cubic spline and threshold effect analyses revealed a positive relationship between PLR and mortality, with an inflection point identified at 4.52. Subgroup analyses further supported the consistency of this association across various clinical subgroups, highlighting PLR as a simple, accessible, and reliable biomarker for early risk stratification in critically ill RHD patients.

Inflammatory biomarkers have become essential tools for prognostic assessment in cardiovascular diseases, where inflammation plays a central role in disease progression and adverse outcomes (14). For example, neutrophils have been shown to mediate the relationship between the cardiovascular metabolic index and all-cause mortality from cardiovascular disease (15). RHD, an autoimmune disorder triggered by group A Streptococcus infection, is characterized by chronic inflammation that leads to progressive valvular damage (16). Several studies have explored the prognostic utility of hematologic inflammatory markers, including PLR, NLR, MLR, SII, and SIRI, in heumatic diseases and related conditions (17–21). In terms of RHD, Giray et al. reported that PLR, NLR, and MLR may aid in the diagnosis and follow-up of ARF, although none effectively reflect the severity of carditis (22). In contrast, other studies have shown that NLR and PLR are positively correlated with the severity of carditis in children with ARF (23), and can reflect disease activity across rheumatic disorders (24). Additionally, In rheumatoid arthritis patients with normal acute phase reactants, the PLR showed a positive correlation with ultrasound-detected synovitis and bone erosion, whereas the NLR and MLR demonstrated no significant association with ultrasonographic findings (25). The superior prognostic performance of PLR in our study aligns with findings in other cardiac and inflammatory conditions, reinforcing its potential clinical relevance.

The biological basis of PLR’s prognostic value in RHD likely stems from the interaction between platelet activation and lymphocyte suppression in the inflammatory and immune responses. Platelets, beyond their role in hemostasis, actively contribute to inflammation through the release of pro-inflammatory cytokines and chemokines (20). In RHD, platelet activation may exacerbate valvular injury by promoting thrombus formation and recruitment of inflammatory cells (26, 27). Similar mechanisms have been observed in coronary artery disease, where platelet-driven inflammation contributes to plaque instability (28, 29). Conversely, lymphocytes, particularly T cells, are key regulators in the autoimmune process of RHD, and lymphopenia reflects immune dysregulation and chronic inflammation (30). An elevated PLR, indicating increased platelet count relative to decreased lymphocyte count, may therefore reflect a pro-inflammatory and immunosuppressive state, a pattern observed in cancer (31) and pulmonary hypertension (32).

Compared with other hematologic markers, PLR may offer a more integrated measure of systemic inflammation. Elevated RDW has been associated with chronic inflammation in pediatric RHD (7), while MPV and PDW indicate platelet activation in cardiovascular diseases (33, 34). However, these single-parameter markers may be less informative than composite indices. Studies in atrial fibrillation (35) and acute myocardial infarction (36) have shown that PLR outperforms NLR and other indices in prognostic accuracy, consistent with our findings. Furthermore, PLR has been linked to multi-organ dysfunction in critically ill patients, suggesting it reflects overall systemic inflammatory burden (37). Our restricted cubic spline analysis demonstrated a positive linear relationship between PLR and 30-day mortality, suggesting that the prognostic value of PLR increases with higher levels, possibly due to intensified platelet-driven inflammation and reduced lymphocyte-mediated immune regulation. As a simple, cost-effective, and routinely available marker, PLR offers practical utility for early risk stratification, especially in resource-limited settings where RHD is prevalent. Future studies should investigate longitudinal changes in PLR and explore its potential as a therapeutic target in RHD.

Although PLR demonstrated superior predictive accuracy over NLR in this study, the independent prognostic value of NLR should not be overlooked. NLR reflects the balance between inflammation and immune regulation, whereas PLR is more indicative of platelet-mediated thrombotic and inflammatory activity (38). The complementary nature of these biomarkers suggests that NLR and PLR may provide distinct yet synergistic insights into the prognostic landscape of RHD (39, 40). The prognostic relevance of NLR likely derives from the divergent roles of neutrophils and lymphocytes in immune responses. Neutrophils, as primary responders to infection and tissue injury, contribute to acute inflammation through the release of cytokines, reactive oxygen species, and proteolytic enzymes (38). In RHD, persistent stimulation by streptococcal antigens leads to chronic neutrophil activation, which promotes ongoing inflammation and valvular remodeling (41). Thus, elevated NLR may reflect an active inflammatory state driving disease progression. Lymphocytes, particularly T cells, are central to the regulation of autoimmune responses in RHD. Dysregulated T-cell subsets, especially imbalances between pro-inflammatory and regulatory T cells, contribute to sustained inflammation and autoantibody production, exacerbating valvular injury (38). A decreased lymphocyte count, reflected by an elevated NLR, may therefore indicate impaired immune regulation and a reduced capacity to resolve inflammation. This combination of enhanced neutrophil-driven inflammation and suppressed lymphocyte-mediated immune control likely underlies the prognostic relevance of NLR in RHD. In contrast, the prognostic value of PLR may stem from the interplay between platelets and neutrophils. Platelets can enhance neutrophil activation through microparticle release, while activated neutrophils promote platelet aggregation, forming a feed-forward loop of inflammation and thrombosis (42). In this context, PLR may better capture thromboinflammatory processes, whereas NLR reflects the balance of innate and adaptive immunity. This mechanistic distinction supports the view that NLR and PLR reflect different dimensions of the inflammatory response in RHD. Future studies should explore the combined utility of NLR and PLR for risk stratification in RHD. Notably, recent evidence suggests that composite indices incorporating both markers are associated with in-hospital mortality in patients with acute myocardial infarction and show enhanced prognostic performance (43, 44).

This study has several limitations. First, its retrospective design and reliance on the MIMIC-IV database preclude definitive conclusions regarding causality between PLR and mortality, and residual confounding may persist despite multivariable adjustment. Due to the high rate of missing values (>40%) for certain laboratory markers (e.g., BNP, lipid profile, liver function tests) and lifestyle factors (e.g., smoking, alcohol use), we were unable to include these variables in our final models. Imputing such data was deemed inappropriate and would risk introducing bias. Second, the data were derived from a single center in the United States, potentially limiting the generalizability of the findings to broader or more diverse RHD populations, particularly in endemic regions. Third, PLR was measured only at ICU admission, and dynamic changes in inflammatory markers during hospitalization were not captured, which may underestimate their full prognostic value. Finally, although PLR outperformed other biomarkers in predicting short-term mortality, the lack of external validation in independent cohorts limits the clinical applicability of these findings.

## Conclusion

This study identifies PLR as a simple, cost-effective, and independent predictor of short-term mortality in critically ill patients with RHD. Its strong prognostic value supports its potential role in early risk stratification. Further prospective multicenter studies are needed to validate these findings and assess the integration of PLR into clinical decision-making frameworks.

## Data Availability

The raw data supporting the conclusions of this article will be made available by the authors, without undue reservation.
