# Supplementary material for: Prognostic value of nine inflammatory biomarkers for critically ill patients with rheumatic heart disease: a retrospective study
Source: Front Immunol. 2025 May 29;16:1610967. doi: 10.3389/fimmu.2025.1610967 (PMC12158921; doi:10.3389/fimmu.2025.1610967)
Supplement: Supplementary file 1 [file DataSheet1.docx]

**Supplementary Materials**

**Supplementary Figure**


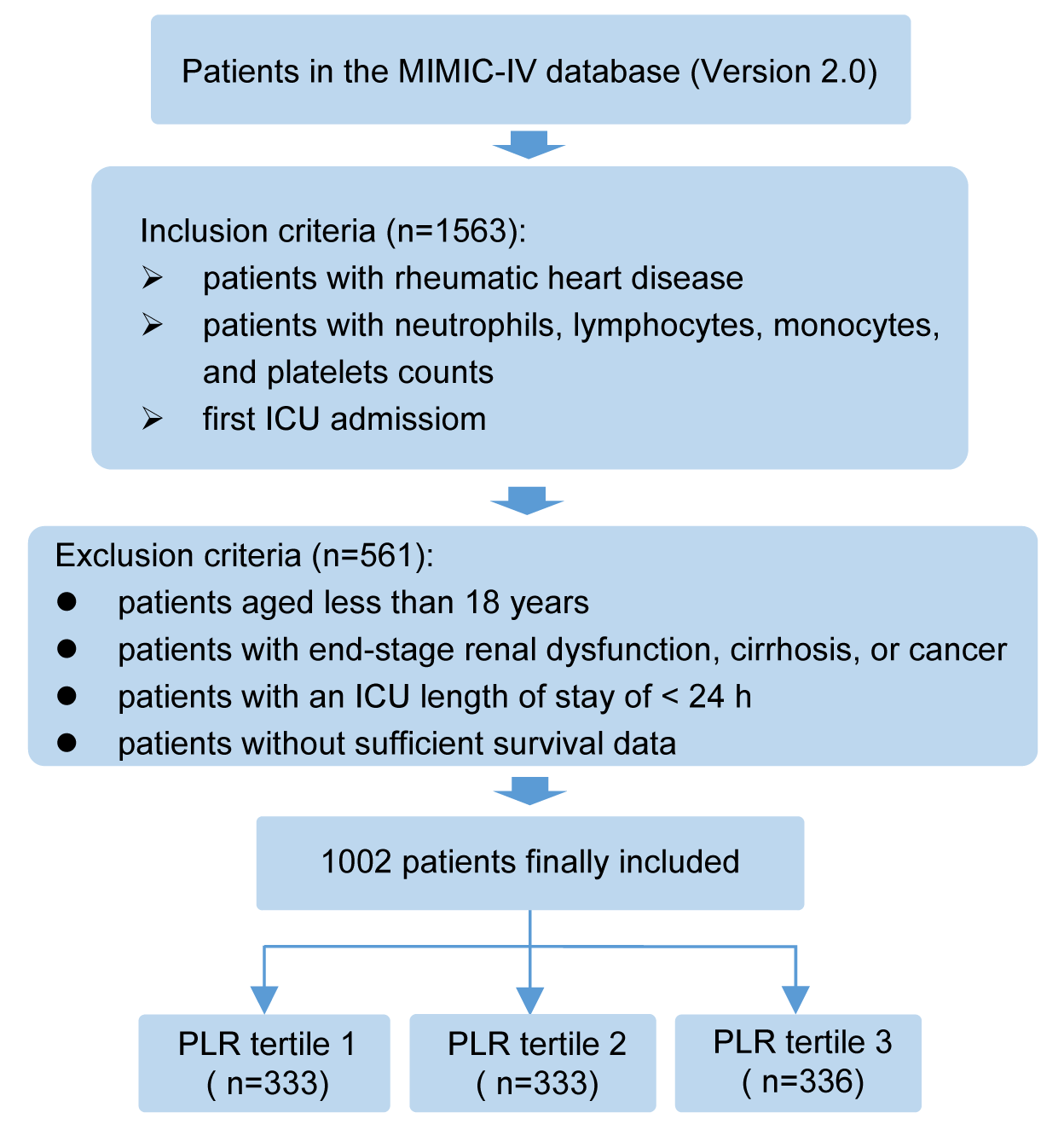


Figure S1. Flow chart of patient selection in this study.

Abbreviation: ICU, intensive care unit; PLRymphocyte ratio.

**Supplementary Tables**

Table S1. Nine inflammatory biomarkers evaluated in this study.

| Indicators | Definition or calculation formula |
| --- | --- |
| NM | neutrophil count (×10^9^) × monocyte count (×10^9^) |
| NP | neutrophil count (×10^9^) × platelet count (×10^9^) |
| MP | monocyte count (×10^9^) × platelet count (×10^9^) |
| NLR | neutrophil count (×10^9^) / lymphocyte count (×10^9^) |
| MLR | monocyte count (×10^9^) / lymphocyte count (×10^9^) |
| PLR | platelet count (×10^9^) / lymphocyte count (×10^9^) |
| SII | platelet count (×10^9^) × neutrophil count (×10^9^) / lymphocyte count (×10^9^) |
| SIRI | neutrophil count (×10^9^) × monocyte count (×10^9^) / lymphocyte count (×10^9^) |
| AIRI | platelet count (×10^9^) × neutrophil count (×10^9^) × monocyte count (×10^9^) / lymphocyte count (×10^9^) |

Abbreviation: AIRI, aggregate index of systemic inflammation response; MLR, monocyte-to-lymphocyte ratio; MP, monocyte × platelet; NLR, neutrophil-to-lymphocyte ratio; NM, neutrophil × monocyte; NP, neutrophil × platelet; PLR, platelet-to-lymphocyte ratio; SII, systemic immune-inflammation index; SIRI, systemic inflammation response index.

Table S2. Missing number for risk variables and outcome variables.

| Variable | Missing number | Percent | Total |
| --- | --- | --- | --- |
| Gender | 0 | 0 | 1002 |
| Age | 0 | 0 | 1002 |
| LOS ICU | 0 | 0 | 1002 |
| 30-day ICU mortality | 0 | 0 | 1002 |
| Heart_failure | 0 | 0 | 1002 |
| Respiratory failure | 0 | 0 | 1002 |
| Diabetes | 0 | 0 | 1002 |
| Paraplegia | 0 | 0 | 1002 |
| Renal disease | 0 | 0 | 1002 |
| Sepsis | 0 | 0 | 1002 |
| Cancer | 0 | 0 | 1002 |
| Hypertension | 0 | 0 | 1002 |
| Rheumatic_heart_disease | 0 | 0 | 1002 |
| Lymphocytes | 0 | 0 | 1002 |
| Monocytes | 0 | 0 | 1002 |
| Neutrophils | 0 | 0 | 1002 |
| Platelet | 60 | 5.99 | 1002 |
| OASIS | 0 | 0 | 1002 |
| GCs | 0 | 0 | 1002 |
| CCI | 0 | 0 | 1002 |

Abbreviation: CCI, Charlson comorbidity index; GCS, Glasgow coma scale; ICU, intensive care unit; LOS, Length of Stay; OASIS, oxford acute severity of illness score.

Table S3. Schoenfeld residual test for the proportional hazards assumption in Cox models of 30-day all-cause mortality among patients with rheumatic heart disease.

| Variable | Model 1 | | Model 2 | | Model 3 | | Model 4 | |
| --- | --- | --- | --- | --- | --- | --- | --- | --- |
|  | chisq | p-value | chisq | p-value | chisq | p-value | chisq | p-value |
| PLR | 1.101 | 0.294 | 1.337 | 0.248 | 0.448 | 0.503 | 0.343 | 0.558 |
| Age |  |  | 1.191 | 0.275 | 1.564 | 0.211 | 0.692 | 0.405 |
| Gender |  |  | 0.019 | 0.891 | 0.001 | 0.981 | 0.011 | 0.915 |
| OASIS |  |  |  |  | 2.041 | 0.154 | 2.658 | 0.326 |
| GCS |  |  |  |  | 0.443 | 0.506 | 0.541 | 0.462 |
| CCI |  |  |  |  | 1.555 | 0.212 | 1.295 | 0.255 |
| Respiratory failure |  |  |  |  |  |  | 0.061 | 0.805 |
| Diabetes |  |  |  |  |  |  | 0.006 | 0.937 |
| Hypertension |  |  |  |  |  |  | 1.106 | 0.293 |
| Renal disease |  |  |  |  |  |  | 2.583 | 0.108 |
| Sepsis |  |  |  |  |  |  | 0.272 | 0.602 |
| Paraplegia |  |  |  |  |  |  | 0.712 | 0.399 |
| Global | 1.101 | 0.294 | 2.071 | 0.558 | 4.568 | 0.062 | 9.312 | 0.139 |

Abbreviation: CCI, charlson comorbidity index; chisq, chi-square statistic; GCS, glasgow coma scale;OASIS, oxford acute severity of illness score; PLR, platelet-to-lymphocyte ratio.
